# Experimental Warming Differentially Influences the Vulnerability of Phototrophic and Heterotrophic Periphytic Communities to Copper Toxicity

**DOI:** 10.3389/fmicb.2018.01424

**Published:** 2018-07-02

**Authors:** Stéphane Pesce, Anne-Sophie Lambert, Soizic Morin, Arnaud Foulquier, Marina Coquery, Aymeric Dabrin

**Affiliations:** ^1^Irstea, UR RiverLy, Centre de Lyon-Villeurbanne, Villeurbanne, France; ^2^Irstea, UR EABX, Centre de Bordeaux, Gazinet-Cestas, France; ^3^UMR CNRS 5553, Laboratoire d’Écologie Alpine, Université Grenoble Alpes, Grenoble, France

**Keywords:** bioaccumulation, biofilms, extracellular enzymatic activities, freshwater, microbial ecotoxicology, multi-stress, photosynthesis, pollution-induced community tolerance (PICT)

## Abstract

Aquatic ecosystems are generally subjected to multiple perturbations due to simultaneous or successive combinations of various natural and anthropogenic environmental pressures. To better assess and predict the resulting ecological consequences, increasing attention should be given to the accumulation of stresses on freshwater ecosystems and its effects on the vulnerability of aquatic organisms, including microbial communities, which play crucial functional roles. Here we used a microcosm study to assess the influence of an experimental warming on the vulnerability of phototrophic and heterotrophic periphytic communities to acute and chronic copper (Cu) toxicity. Natural periphytic communities were submitted for 4 weeks to three different temperatures (18, 23, and 28°C) in microcosms contaminated (at about 15 μg L^-1^) or not with Cu. The vulnerability of both phototrophic and heterotrophic microbial communities to subsequent acute Cu stress was then assessed by measuring their levels of sensitivity to Cu from bioassays targeting phototrophic (photosynthetic activity) and heterotrophic (β-glucosidase and leucine aminopeptidase extracellular enzymatic activities) microbial functions. We postulated that both the increase in temperature and the chronic Cu exposure would modify microbial community structure, thus leading to changes in the capacity of phototrophic and heterotrophic communities to tolerate subsequent acute exposure to Cu. Our results demonstrated that the influence of temperature on the vulnerability of phototrophic and heterotrophic microbial communities to Cu toxicity can vary greatly according to function studied. These findings emphasize the importance of considering different functional compartments and different functional descriptors to better assess the vulnerability of periphyton to multiple stresses and predict the risks induced by multiple stressors for ecosystem balance and functioning.

## Introduction

Metals are ubiquitously present in freshwater ecosystems, whether occurring naturally (Bradl, 2005) or anthropogenically due to releases from agriculture, urbanization, mining, and industry (Ancion et al., 2013). Copper (Cu) is well representative of the widespread metal pollution of surface waters including rivers and streams in agricultural areas, where it has been widely used as a fungicide and weed-killer in both conventional and organic agriculture (Serra and Guasch, 2009; Provenzano et al., 2010). Cu is an essential element for organisms, serving as a cofactor in many enzymatic pathways that catalyze a wide variety of functions including several redox reactions as well as photosynthetic and mitochondrial electron transport (Ladomersky and Petris, 2015; Adams et al., 2016). However, high concentrations of Cu are toxic to aquatic life, and thus pose ecotoxicological risks in freshwater ecosystems. There is a large ecotoxicological dataset on the effects of Cu on aquatic macroorganisms, especially fish and invertebrates (e.g., US EPA, 2010). Aquatic microbial communities, which are composed of phototrophic and heterotrophic microorganisms (including microalgae, bacteria, fungi, and heterotrophic protists) can also be impacted by Cu toxicity, which can generate excess reactive oxygen species (Okamoto et al., 2001; Sabatini et al., 2009), induce lipid peroxidation (Rijstenbil et al., 1994), increase membrane permeability (Cid et al., 1995), inhibit growth (Prasad et al., 1998), and reduce chlorophyll or accessory pigment contents (Rijstenbil et al., 1994).

In lotic ecosystems including agricultural streams, benthic microbial assemblages such as periphytic communities (also called “biofilms”) provide key ecological functions, like primary production and nutrient recycling (Battin et al., 2003). These microbial communities, which are embedded in a polysaccharide–protein matrix, quickly interact with dissolved chemicals (Sabater et al., 2007), including Cu. chronic exposure to Cu can functionally impact phototrophic and heterotrophic periphytic communities by reducing photosynthesis (Lambert et al., 2012), extracellular enzymatic activities such as β-glucosidase activity (Lambert et al., 2012) and substrate-induced respiration (Tlili et al., 2011). It can also modify community structure following changes in microbial biomasses, distribution of algal classes, taxonomic composition of diatom communities (Serra and Guasch, 2009; Morin et al., 2017), and bacterial diversity (Tlili et al., 2010). Microorganisms have nevertheless developed several defense mechanisms to cope with Cu toxicity (see Nies, 1999; Bruins et al., 2000 for details). The main mechanisms of Cu resistance include efflux ATPase pumps able to throw out Cu ions, chemo-osmotic Cu extrusion systems such as the cus system encoding especially for the CusA protein belonging to the resistance, nodulation, and cell division family responsible for metal export, and the periplasmic pco system unique to plasmids (Besaury et al., 2013). The polysaccharide–protein matrix, which plays a crucial structural role in biofilms, also serves as a protective layer protect against environmental stress such as metallic contamination due to its high metal ion adsorption capacity, which can prevent their diffusion to deeper layers of the periphyton, thus reducing microbial community exposure (Rose and Cushing, 1970; Loaec et al., 1997; Lau et al., 2005).

The combination of structural changes (which are generally substantially attributable to the loss of sensitive species and/or the development of more tolerant ones) and microbial adaptation at individual level following chronic exposure to Cu can increase the potential of both phototrophic and heterotrophic periphytic microbial communities to tolerate Cu (Soldo and Behra, 2000; Tlili et al., 2010; Lambert et al., 2012), as postulated by the concept of pollution-induced community tolerance (PICT) first introduced by Blanck et al. (1988). Indeed, such adaptation processes partly condition the resilience and resistance capacities of microbial communities following acute or chronic exposure to Cu, thus defining their vulnerability to this toxicant.

Given the fact that aquatic microbial communities are generally subjected to multiple natural and anthropogenic environmental stressors due to simultaneous or successive combinations of various pressures (e.g., pollution, physical stresses, competition, and predation), it is necessary to better assess and predict how communities respond to multiple stressors rather than just a single compound (Clements and Rohr, 2009; Holmstrup et al., 2010). Accordingly, research needs to focus more attention on the accumulation of stresses on freshwater ecosystems and its resulting effects on the vulnerability of aquatic communities, including microbial ones (Morin et al., 2015).

Due to global warming, many freshwater ecosystems are increasingly subjected to extreme climate events including prolonged high-temperature periods (Easterling et al., 2000; Wreford and Adger, 2010). The resulting acute increase in water temperature can represent a heat stress that can interact with the toxicant effects induced by Cu exposure. Indeed, communities already sensitized by a temperature rise would likely be impoverished in terms of species and/or functionalities and consequently lose some capacity to cope with an additional stress such as toxicant exposure (e.g., Morin et al., 2015). While most of the studies dealing with the combined effects of heat stress and metal exposure on aquatic populations have concerned macroorganisms (for a review, see Holmstrup et al., 2010), there is increasing evidence that temperature conditions influence periphytic microbial community response to Cu toxicity (Boivin et al., 2005; Lambert et al., 2016, 2017; Morin et al., 2017). For instance, experimental warming (+5 to +15°C) was shown to significantly influence the chronic and acute ecotoxicological impact of Cu on phototrophic periphytic communities by reducing Cu bioavailability and bioaccumulation in periphyton (Lambert et al., 2016), inducing changes in diatom communities (Lambert et al., 2016; Morin et al., 2017), and decreasing both the basal and Cu-induced tolerance of phototrophic communities to acute Cu exposure (Lambert et al., 2017; Morin et al., 2017). Focusing on bacterial communities, Boivin et al. (2005) also highlighted the potential effect of temperature on PICT processes. However, and contrary to Lambert et al. (2017) and Morin et al. (2017), they showed a gradual increase in Cu-induced community tolerance when temperature increased from 10 to 20°C (Boivin et al., 2005). Interestingly, this experimental observation was consistent with the findings of a field survey by Faburé et al. (2015) who showed that heterotrophic periphytic communities collected in winter were less Cu-tolerant than those collected in warmer seasons.

Taken together, these studies reveal that temperature can greatly influence the vulnerability of phototrophic and heterotrophic periphytic communities to Cu toxicity. However, results were quite divergent among studies, suggesting that the magnitude and direction of this influence depend on several parameters, such as magnitude of both Cu and temperature stresses and initial community composition which condition the sensitivity to Cu of the most thermotolerant species (Lambert et al., 2016), and the kind of microbial communities involved (i.e., phototrophic or heterotrophic). Moreover, until now, the combined effects of temperature and Cu on both the phototrophic and heterotrophic components of periphytic communities have not been simultaneously investigated.

In this context, research is still needed to better understand the mechanisms that drive the influence of temperature on the vulnerability of phototrophic and heterotrophic periphytic communities to acute and chronic Cu toxicity. To address this issue, we used a microcosm study where natural periphytic communities were submitted for 4 weeks to three different temperatures (18, 23, and 28°C) in microcosms contaminated (at about 15 μg L^-1^) or not with Cu. For each treatment, we assessed the vulnerability of both phototrophic and heterotrophic microbial communities to subsequent acute Cu stress by measuring their levels of sensitivity to Cu via bioassays targeting phototrophic (photosynthetic activity) and heterotrophic (extracellular enzymatic activities) microbial functions. Given the potential influence of temperature on Cu bioavailability and accumulation in periphytic assemblages (Lambert et al., 2016) and the important protective role of the polysaccharide–protein matrix to Cu exposure, we also focused effort on measuring Cu concentrations in (i) the colloidal, (ii) the capsular fractions of the extracellular polymeric substances (EPS), and (iii) in the intracellular fraction of periphyton. In addition, we assessed structural changes in periphyton by measuring algal biomass and photosynthetic efficiency and by performing diatom taxonomic analysis.

Besides the simultaneous study of phototrophic and heterotrophic communities, the main originality of the study lies in the fact that we considered Cu and temperature stress levels representative of point-in-time contaminations and thermal conditions observed in the downstream section of the river where the periphytic communities were initially collected. We postulated that both the increase in temperature and the chronic Cu exposure would modify microbial community structure, thus leading to changes in the capacity of phototrophic and heterotrophic communities to tolerate subsequent acute exposure to Cu. In line with the PICT concept, our first hypothesis was that chronic exposure to Cu would have made both phototrophic and heterotrophic communities more tolerant to acute Cu toxicity. Based on the literature cited above, our second hypothesis was that the increase in temperature would have increased the vulnerability of phototrophic communities and decreased the vulnerability of heterotrophic communities to acute Cu toxicity, whatever their history of chronic Cu exposure.

## Materials and Methods

### Experimental Setup

**FIGURE 1:**
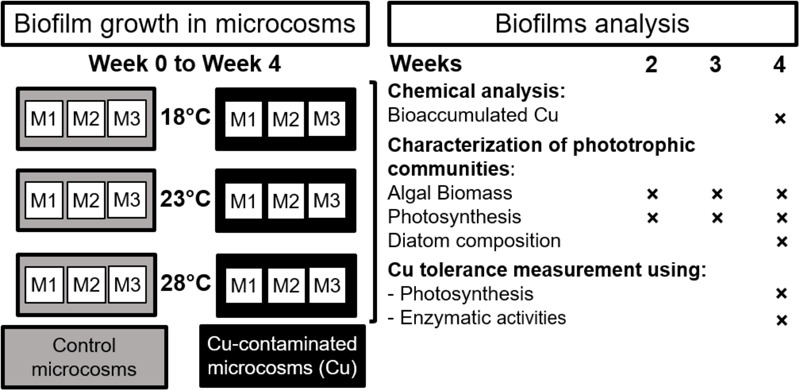
Microcosm experimental design and analytical strategy. *M: Independent microcosm*.

The experiment was carried out using a natural biofilm inoculum collected from the Morcille river (Beaujolais, Eastern France) in September 2014. Stones were collected at the upstream reference site of the Morcille River (see Rabiet et al., 2015 for details). The periphyton was scraped and suspended in the river water in order to obtain a periphytic inoculum, which was homogenized before being added at the start of the experiment (week 0) for colonization of glass slides vertically immersed a few centimeters below the water surface in the microcosms. The microcosms consisted in 18 independent glass aquariums (40 × 20 × 25 cm) incubated in three polyethylene tanks (250 L, 121 × 81 × 33 cm) containing water thermoregulated at 18°C (i.e., close to the average seasonal temperature of Morcille river water during the periphyton sampling), 23 and 28°C, respectively (**Figure 1**). This experimental warming can be considered as a realistic worst-case scenario, as recorded in the Morcille river in summer 2015 (e.g., 26.9°C recorded on July 21, 2015, at 6:00 p.m. on the downstream section of the river, unpublished data).

In each tank, six microcosms were filled with reconstituted water consisting of 3:1 (v/v) distilled water:groundwater, supplemented with nutrients in order to adjust conductivity (i.e., about 180 μS cm^-1^) and nutrient concentrations (i.e., 15 mg L^-1^ of silica; 8 mg L^-1^ of nitrates; 0.2 mg L^-1^ of orthophosphates) to the characteristics of the river water at the periphyton sampling site (**Table 1**). Water mixing, oxygenation, and lighting were operated as previously described in Lambert et al. (2015). Three microcosms were used as Control microcosms (“Control,” without Cu addition) and three Cu-exposed microcosms (“Cu”) were supplemented with CuSO_4_, 5H_2_O to obtain a Cu concentration close to the highest concentrations recorded in the downstream section of Morcille River (i.e., about 15 μg Cu L^-1^; Montuelle et al., 2010; Rabiet et al., 2015). To avoid Cu adsorption by the experimental equipment during the exposure period, Cu microcosms (including glass slides and pumps) were saturated using the same Cu concentration for 24 h before start of the experiment.

**Table 1:** Physico-chemical characteristics of water in the six tested conditions before (conditions after 1 week) and 2 h after (initial conditions) each water renewal during 4 weeks (mean values of weekly samples ± SD; *n* = 3).

	Initial conditions (after water renewal)	Conditions after 1 week (before water renewal)					
		18°C		23°C		28°C	
	**All aquariums (*n* = 18)**	**Control (*n* = 3)**	**Cu (*n* = 3)**	**Control (*n* = 3)**	**Cu (*n* = 3)**	**Control (*n* = 3)**	**Cu (*n* = 3)**

pH	9.0 ± 0.1	8.9 ± 0.4	9.4 ± 0.4	9.0 ± 0.4	9.0 ± 0.7	8.9 ± 0.3	8.9 ± 0.4
Conductivity (μS cm^-1^)	130 ± 6	118 ± 17	110 ± 17	113 ± 12	122 ± 23	118 ± 13	120 ± 22
Dissolved O_2_ (mg L^-1^)	9.25 ± 0.69	10.65 ± 0.30	11.04 ± 0.62	9.84 ± 0.40	10.24 ± 0.85	9.39 ± 0.38	9.83 ± 0.81
Dissolved Organic C (mg L^-1^)	0.58 ± 0.24	2.22 ± 0.41	1.89 ± 0.37	2.08 ± 0.50	1.88 ± 0.50	2.13 ± 0.47	1.73 ± 0.46
NO_3_ (mg L^-1^)	5.81 ± 0.45	1.28 ± 0.43	1.10 ± 0.20	1.30 ± 0.51	2.01 ± 1.57	1.57 ± 0.87	1.66 ± 1.61
NO_2_ (mg L^-1^)	<0.05	<0.05	<0.05	0.06 ± 0.01	0.14 ± 0.14	0.06 ± 0.02	0.14 ± 0.16
NH_4_ (mg L^-1^)	0.03 ± 0.04	0.05 ± 0.10	<0.02	<0.02	<0.02	<0.02	<0.02
PO_4_ (mg L^-1^)	0.15 ± 0.05	<0.10	<0.10	<0.10	<0.10	<0.10	<0.10
SiO_2_ (mg L^-1^)	5.53 ± 0.33	0.64 ± 0.55	2.27 ± 1.75	0.91 ± 0.67	1.89 ± 1.52	1.47 ± 1.00	4.11 ± 2.25

This study was conducted for 4 weeks. After 1 week, the water level of each aquarium was adjusted to its initial value, and nutrients were added to maintain the initial trophic conditions. Then, water was renewed weekly to maintain Cu exposure and avoid nutrient depletion.

### Main Physico-Chemical Analyses

The main physical–chemical parameters of the water were measured in each microcosm before and 2 h after each water renewal. Conductivity, pH, and dissolved oxygen concentration were measured using portable meters (WTW). Water samples were collected at the same time for subsequent laboratory analyses. Standard operating procedures were followed to determine the concentrations of orthophosphates (PO_4_), nitrates (NO_3_), nitrites (NO_2_), ammonium (NH_4_), silicon dioxide (SiO_2_), and dissolved organic carbon (DOC), as described in Lambert et al. (2017). Temperature was recorded every hour with dataloggers (HOBO^®^ Pendant Temperature/Light, Prosensor).

### Copper Analysis in Water and in Periphyton

Total dissolved Cu concentrations were measured before and after each water renewal. Channel water (30 mL) was sampled, filtered [0.45 μm polyvinylidene difluoride (PVDF), Whatman], acidified to 0.5% (v/v) with nitric acid (14 M Suprapur, Merck), and stored at 4°C until analysis.

Cu concentrations in periphyton were measured at week 4 following a protocol adapted from Aguilera et al. (2008) to determine the concentrations of Cu in (i) the colloidal and (ii) the capsular fraction of the biofilm EPS as well as in (iii) the intracellular fraction of periphyton. Periphyton was scraped from glass substrates and suspended in the reconstituted water used to fill the microcosms. After homogenization, 30 mL of the obtained biofilm suspension was lyophilized. Demineralized water (10 mL) was then added to obtain a suspension, which was gently shaken during 20 min at ambient temperature and centrifuged (4000 *g*, 15 min) to retrieve the supernatant containing the colloidal EPS fraction. Then, the pellet was incubated with EDTA (final concentration 4 mM) during 3 h under shaking at ambient temperature, and centrifuged (14,000 *g*, 20 min) to retrieve the supernatant containing the capsular EPS fraction. Both colloidal and capsular EPS fractions were acidified with 0.5% (v/v) nitric acid (14 M Suprapur, Merck) and stored at 4°C until analysis. The final pellet was dried and mineralized with nitric acid (14 M) in a microwave oven (CEM, Mars-5) to extract the intracellular Cu fraction.

Total dissolved Cu samples were analyzed by inductively coupled plasma–atomic emission spectroscopy (ICP*-*AES, Agilent 720-ES) and inductively coupled plasma–mass spectrometry (ICP-MS Series II, Thermo Electron). Periphyton extracts were analyzed by ICP-MS. Limits of quantification (LQ) were 1 μg L^-1^ in water samples and 3 μg g^-1^ dry weight (dw) in biofilms for ICP-AES, and 0.050 μg L^-1^ and 1.5 μg g^-1^ dw for ICP-MS. Routine quality Control checks used a certified reference material (River water, TM 27.3; plankton, BCR-414).

### Periphyton Characterization

After 14, 21, and 28 days in the microcosms, periphyton was carefully scraped from the slides with a plastic spatula and suspended in 1:1 (v/v) demineralized:mineral water (Volvic) before being homogenized and aliquoted to further measure the following parameters. Total chlorophyll *a* (chl *a*) and photosynthetic efficiency (derived from the maximal PSII quantum yield) were estimated weekly by multi-wavelength pulse-amplitude-modulated (PAM) fluorometry using a Phyto-PAM system (H. Walz GmbH) as described in Lambert et al. (2017). Diatom identification was performed on the inoculum and at week 4 as described in Morin et al. (2017).

### Tolerance Assessment of Phototrophic and Heterotrophic Communities to Copper (PICT Measurement)

A semi-logarithmic series of Cu concentrations was freshly prepared in 1:1 (v/v) demineralized:mineral water (Volvic) to obtain seven different Cu concentrations ranging from 0.32 to 320 mg/L for phototrophic tolerance assessment and from 0.032 to 32 mg/L for heterotrophic tolerance assessment. Cu concentrations in each dilution were checked by ICP-MS (ICP-MS X Series II, Thermo Electron) as described above.

Phototrophic and heterotrophic periphyton community tolerance to Cu was assessed at week 4 using photosynthetic efficiency, based on the measurement of maximal quantum yield (*Y*_II_), as endpoint for phototrophs and β-glucosidase (β-Glu), leucine aminopeptidase (Lap), and phosphatase (Pase) activities as endpoints for heterotrophs.

Given the influence of periphyton biomass on PICT measurement (Lambert et al., 2015), the initial periphyton suspension was diluted with the demineralized:mineral water mixture before toxicity tests. Based on the assumption that phototrophic biomass (estimated from chl *a* concentrations measured with the Phyto-PAM fluorometer) can be viewed as a proxy of total biomass of phototrophic biofilms (Lambert et al., 2015), periphyton suspensions were diluted to a standardized chl *a* concentration for further assessment of phototrophic and heterotrophic tolerance levels.

To assess phototrophic community tolerance, diluted periphyton suspensions (1.8 mL, at about 2000 μg chl *a*/L) were exposed to increasing concentrations of Cu (0.9 mL) in a climate chamber (MLR-350 Versatile Environmental Test Chamber, Sanyo) at the intermediate temperature of 23°C (Lambert et al., 2017) under artificial light (1400 lux). Samples were then kept for 30 min in a dark chamber, and PSII quantum yield (665 nm) was determined as stated above.

For heterotrophic communities, β-Glu, Lap, and Pase activities were measured using fluorescent-linked substrates [4-methylumbelliferyl-β-D-glucopyranoside (MUF-GLU), L-leucine-7-amido-4-methylcoumarin hydrochloride (MCA-Lap) and 4-methylumbelliferyl phosphatase (MUF-P); Sigma-Aldrich]. First, diluted periphyton suspensions (1.7 mL, at about 5000 μg chl *a*/L) were exposed to increasing concentrations of Cu (0.3 mL) in the dark, at 23°C, under gentle shaking, for 1 h. Then, periphyton suspensions were incubated with 1 mL of MUF-GLU, MCA-Lap, or MUF-P at saturating concentrations (750 μM for MUF-Glu and MCA-Lap and 3000 μM for MUF-P), in the dark, at 23°C, under gentle shaking, for 1h40 (β-Glu), 1h (Lap), or 1h30 (Pase). Then, 300 μL of glycine buffer (pH 10.4; glycine 0.05 M, NH_4_OH 0.2 M) was added to stop the enzymatic reaction. Fluorescence of MUF and MCA was measured promptly following centrifugation (5000 *g*, 10 min) using a Biotek SynergyHT fluorometer at 360/460 nm excitation/emission. For each sample replicate, four blanks and two analytical replicates were analyzed for each concentration.

### Data Processing

Variations in dissolved Cu among thermal conditions and sampling dates and variations in water physico-chemical characteristics among thermal and treatment conditions were tested using two-way repeated measures ANOVA followed by a *post hoc* Tukey test in R version 2.15.0 (R Development Core Team, 2012). Data were log-transformed before statistical analysis to satisfy the assumption of normality and homogeneity of variances. Variations in Cu bioaccumulation (colloidal, capsular, and intracellular fractions) in the Cu-exposed biofilms among thermal conditions were tested using one-way repeated measures ANOVA followed by a *post hoc* Tukey test.

PICT bioassay results were analyzed using functions from the “drc” package (Ritz and Streibig, 2005) in R version 2.15.0 (R Development Core Team, 2012). Dose–response curves were fitted to the data using the four-parameter log-logistic model given by the formula:

$$\text{response}=c+\frac{d-c}{1+\exp\left\{b\times \left(\log\left(\text{Dose}\right)-\log\left(e\right)\right)\right\}}$$

where *b* is the slope of the curve around *e*, parameters *c* and *d* are the lower and upper limits of the curve, respectively, and *e* is EC_50_, i.e., the dose producing a response half-way between the upper and the lower limit. EC_50_ variations among thermal and treatment conditions were then tested using two-way repeated measures ANOVA followed by a *post hoc* Tukey test. Significance for all statistical tests was set at *p* < 0.05.

## Results

### Physico-Chemical Data

Water temperatures measured every hour in each microcosm were close to target temperatures with mean values of 18.3°C (±0.2), 23.5°C (±0.3), and 28.0°C (±0.2), respectively, without significant difference between Control and Cu microcosms (*p* > 0.05). **Table 1** reports the mean values of the main physical–chemical variables measured in the microcosms before and after each water renewal. In both Control and Cu-exposed microcosms, dissolved oxygen concentrations decreased significantly from 10.6 to 9.8 mg L^-1^ between 18 and 28°C (*p* < 0.05). Silica concentrations decreased over time, but to a lower extent with higher temperatures (*p* > 0.05). Silica concentrations were higher in Cu microcosms for each temperature tested, and the difference was significant at 28°C (*p* < 0.05). In contrast, DOC concentrations slightly decreased in Cu-exposed microcosms compared to Control microcosms (*p* > 0.05).

### Dissolved Copper Concentrations

In Control microcosms (i.e., without Cu addition), dissolved Cu concentrations remained below the limit of detection throughout the experiment. In all Cu-exposed microcosms, mean dissolved Cu concentrations measured 2 h after water renewal were 10.2 ± 0.4 μg L^-1^ at week 2 and 8.3 ± 0.2 μg L^-1^ at week 3, without significant difference between temperatures, whatever the sampling time (*p* > 0.05; data not shown). Dissolved Cu concentrations were relatively stable in the microcosms between each water renewal (*p* > 0.05) without a significant effect of temperature on Cu concentrations recorded one week after water renewal (*p* > 0.05).

### Copper Bioaccumulation

**FIGURE 2:**
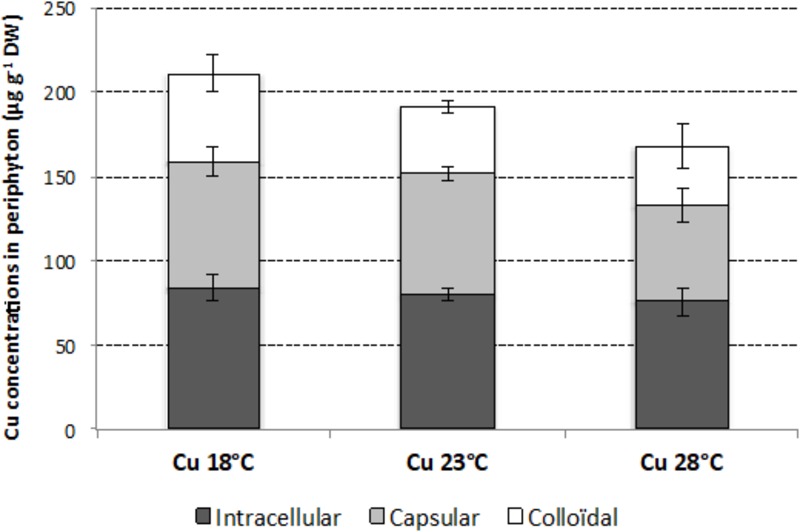
Mean (±SD; *n* = 3) intracellular, capsular, and colloidal concentrations of Cu in periphyton (μg g^-1^ dry weight) at week 4, in the Cu-exposed microcosms incubated at 18, 23, and 28°C.

After 4 weeks of exposure, total Cu concentration in periphyton growing in Cu-exposed microcosms was 211 ± 11 μg g^-1^ dw at 18°C, 192 ± 2 μg g^-1^ dw at 23°C, and 168 ± 25 μg g^-1^ dw at 28°C, with no significant difference between temperatures (*p* > 0.05), despite a tendency of Cu accumulated to decrease with increasing temperatures (**Figure 2**). This tendency was mainly due to a decrease in externalized Cu concentrations, represented by colloidal and capsular fractions. In contrast, internalized Cu concentrations, represented by the intracellular fraction, were relatively stable, with mean values close to 80 μg g^-1^ dw, whatever the temperature.

### Periphyton Characterization

#### Algal Biomass and Photosynthetic Efficiency

**FIGURE 3:**
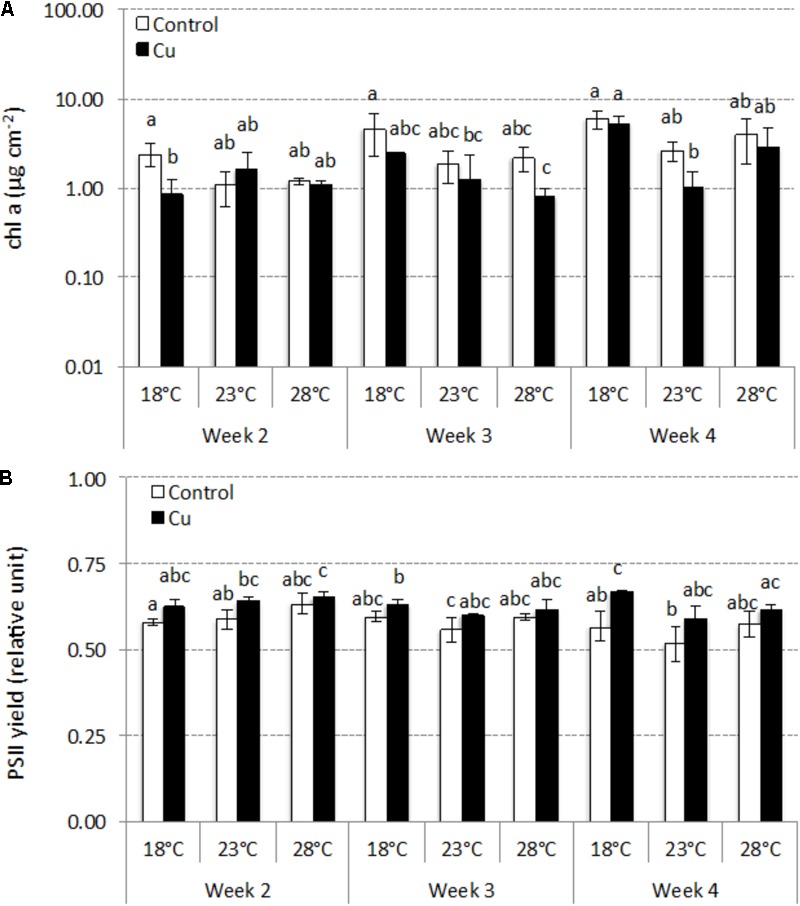
Time-course of concentrations of chlorophyll a (mean ± SD, μg cm^-2^; *n* = 3) **(A)** and PSII yield (mean ± SD, relative units; *n* = 3) **(B)** between week 2 and week 4 in Control and Cu-exposed microcosms incubated at 18, 23, and 28°C. Different letters indicate significant differences between treatments (thermal and exposure context) at a given sampling time (ANOVA, *p* < 0.05).

Applied individually, each stress (i.e., warming or chronic Cu exposure) had a limited effect on chl *a* concentrations (**Figure 3A**). In Control microcosms (i.e., without Cu stress), chl *a* concentrations gradually increased during the 4 weeks, without significant difference between temperatures (*p* > 0.05; **Figure 3A**). At 18°C (i.e., without warming stress), chronic Cu exposure had only a transient negative effect at week 2 (*p* < 0.05). The combined effects of warming and Cu were more pronounced and led to a significant decrease (*p* < 0.05) in chl *a* at week 3 (23°C–Cu and 28°C–Cu) and week 4 (23°C-Cu) compared to 18°C–Control microcosms.

The warming stress alone had no effect on photosynthetic efficiency, which remained stable during 4 weeks in Control microcosms (0.58 ± 0.03) without significant difference between temperatures (*p* > 0.05; **Figure 3B**). Throughout the experiment and for each temperature tested, photosynthetic efficiency was slightly higher in Cu-exposed communities (0.63 ± 0.02), although the different only reached significance after 4 weeks at 18°C (*p* < 0.05). The combined effect of warming and Cu led to a significant, but only transient, increase of photosynthetic efficiency at week 2 in both 23°C–Cu and 28°C–Cu microcosms (*p* < 0.05) compared to 18°C–Control microcosms.

#### Diatom Assemblage Structure

The initial diatom assemblage (i.e., inoculum) sampled in the Morcille River was characterized by a higher specific richness (31 ± 3) than those sampled in the microcosms after a 4-week growing period (7 to 20 species according to temperature and Cu exposure). The 17 dominant diatom species (occurring at more than 3% relative abundance in at least one sample, including initial inoculum) are listed in **Table 2**. The initial diatom assemblage was dominated by three main species (i.e., *Cocconeis pseudolineata, Cocconeis placentula, Rhoicosphenia abbreviata*), which each represented >10% of total diatom assemblage.

**Table 2:** Mean relative abundances (±SD; *n* = 3) of the 17 dominant diatom species (i.e., representing more than 3% relative abundances in at least one sample) for the inoculum and the six thermal and exposure contexts at week 4.

	Inoculum	18°C		23°C		28°C	
		Control	Cu	Control	Cu	Control	Cu
*Achnanthidium minutissimum*	8.6 ± 2.3	17.7 ± 5.3	3.2 ± 1.0	1.4 ± 0.5	0.5 ± 0.5	0.5 ± 0.3	0.6 ± 0.3
*Amphora inariensis*	7.4 ± 1.8	0.0 ± 0.0	0.1 ± 0.2	0.0 ± 0.0	0.0 ± 0.0	0.1 ± 0.2	0.0 ± 0.0
*Amphora pediculu*	5.4 ± 1.3	0.2 ± 0.3	0.1 ± 0.2	0.0 ± 0.0	0.0 ± 0.0	0.0 ± 0.0	0.0 ± 0.0
*Cyclotella comensis*	0.0 ± 0.0	1.8 ± 0.5	0.0 ± 0.0	1.3 ± 0.2	0.1 ± 0.2	0.0 ± 0.0	0.0 ± 0.0
*Cyclotella sp.*	0.0 ± 0.0	4.2 ± 2.1	0.2 ± 0.1	3.3 ± 0.0	0.0 ± 0.0	0.3 ± 0.4	0.0 ± 0.0
*Cocconeis pseudolineata*	15.6 ± 5.3	0.1 ± 0.1	0.0 ± 0.0	0.1 ± 0.3	0.2 ± 0.3	0.0 ± 0.0	0.1 ± 0.2
*Cocconeis placentula var. lineata*	11.4 ± 1.8	0.8 ± 1.0	1.0 ± 0.8	0.5 ± 0.4	1.1 ± 0.4	0.1 ± 0.1	0.1 ± 0.2
*Eolima minima*	5.7 ± 2.3	1.2 ± 0.6	2.4 ± 0.7	1.6 ± 0.1	0.9 ± 0.1	0.1 ± 0.2	2.3 ± 0.9
*Fragilaria gracilis*	0.5 ± 0.5	32.7 ± 15.1	5.7 ± 3.3	63.9 ± 2.2	7.8 ± 2.2	97.0 ± 2.5	11.6 ± 7.4
*Gomphonema exilissimum*	0.0 ± 0.0	3.4 ± 4.0	0.0 ± 0.0	3.4 ± 0.3	0.3 ± 0.3	0.0 ± 0.0	0.0 ± 0.0
*Mayamaea permitis*	0.4 ± 0.3	5.5 ± 3.6	0.9 ± 1.3	1.3 ± 0.1	0.2 ± 0.1	0.0 ± 0.0	0.0 ± 0.0
*Navicula gregaria*	2.2 ± 1.0	0.0 ± 0.0	0.0 ± 0.0	0.0 ± 0.0	0.0 ± 0.0	0.2 ± 0.2	0.0 ± 0.0
*Nitzschia linearis*	0.0 ± 0.0	0.5 ± 0.2	1.8 ± 1.3	0.5 ± 1.2	2.7 ± 1.2	0.0 ± 0.0	0.5 ± 0.6
*Nitzschia palea*	1.8 ± 0.4	26.4 ± 12.5	75.6 ± 10.1	15.3 ± 4.3	83.1 ± 4.3	0.8 ± 1.4	80.9 ± 8.4
*Planothidium frequentissimum*	3.2 ± 0.8	0.2 ± 0.1	0.3 ± 0.3	0.2 ± 0.2	0.1 ± 0.2	0.0 ± 0.0	0.2 ± 0.2
*Planothidium lanceolatum*	7.7 ± 1.6	1.1 ± 0.4	5.1 ± 3.5	1.3 ± 0.8	1.2 ± 0.8	0.0 ± 0.0	0.0 ± 0.0
*Rhoicosphenia abbreviata*	11.9 ± 1.9	0.0 ± 0.0	0.0 ± 0.0	0.2 ± 0.0	0.0 ± 0.0	0.2 ± 0.2	0.0 ± 0.0

Applied individually, the warming stress led to a significant decrease in specific richness but only at 28°C. In Control microcosms, species richness was similar between 18 and 23°C (20 ± 0) but was threefold lower at 28°C (7 ± 1). After 4 weeks at 18°C, Control communities were dominated by *Fragilaria gracilis* (FGRA) and *Nitzschia palea* (NPAL), which represented about 33 and 26%, respectively, of total relative diatom abundance. Under warming stress alone, the proportion of NPAL and FGRA was modified, with a gradual increase in proportion of FGRA which became strongly dominant at 23°C (63.9 ± 2.3%) and especially at 28°C (97.0 ± 2.5%), while NPAL followed a reverse trend, accounting for less than 3% of total diatoms at 28°C.

Cu stress alone exerted the opposite effect, inducing an increase in proportion of NPAL and a decrease in proportion of FGRA, which represented about 76 and 6%, respectively, of total relative diatom abundance at week 4. Under Cu exposure, no drastic change was observed with increasing temperature, as illustrated by both the relative abundance of these two dominant diatom species and specific richness (14 ± 3) which were relatively similar at the three temperatures tested.

The proportion of *Achnanthidium minutissimum* (ADMI), which was well represented at 18°C in the Control communities (17.7 ± 5.3%), significantly decreased under the two kinds of stresses, both alone and in combination.

#### Tolerance Assessment

##### Phototrophic communities

**FIGURE 4:**
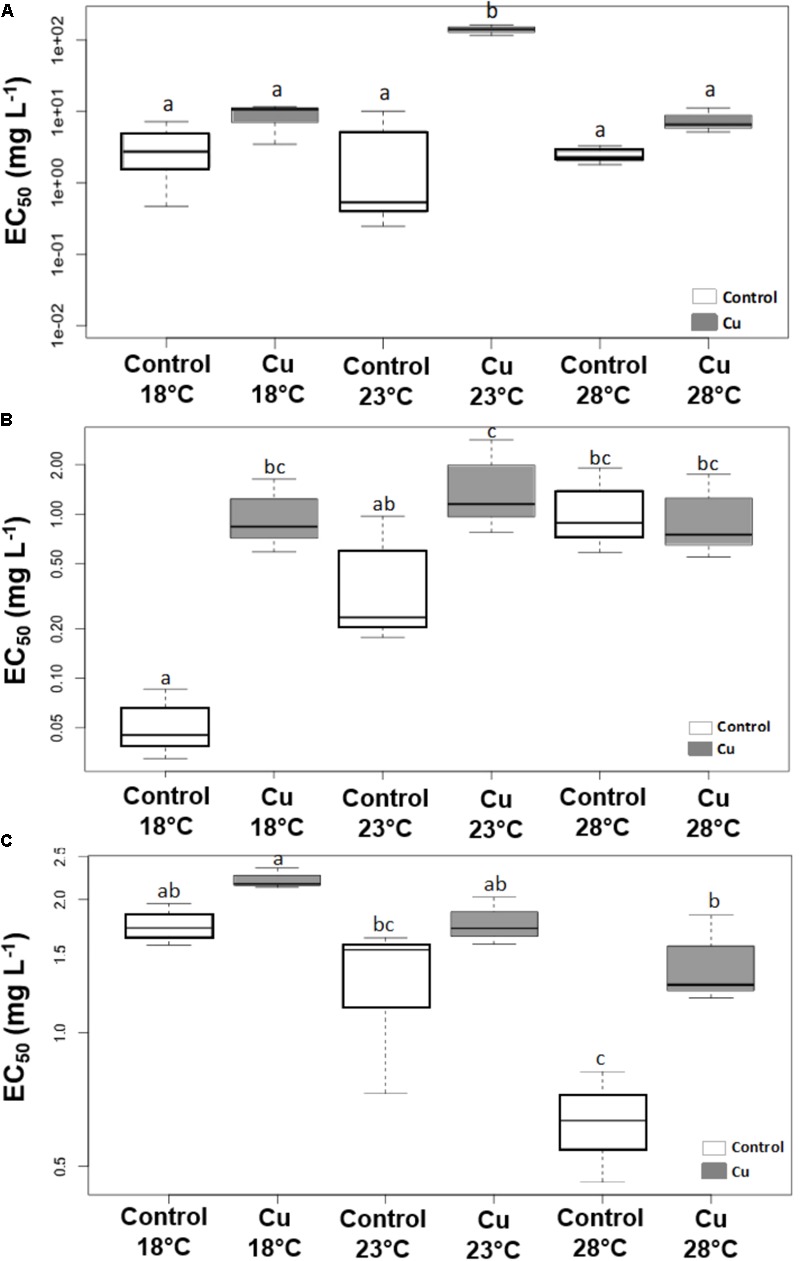
Boxplot of EC_50_ values (mg Cu L^-1^) determined at week 4 for phototrophic communities based on photosynthetic yield **(A)** and for heterotrophic communities based on β-glucosidase **(B)** and Leucine aminopeptidase **(C)** activities. The horizontal line represents the median. The upper and lower limits of the box represent the 75th and 25th percentiles. Different letters indicate significant differences between treatments (ANOVA, *p* < 0.05, *n* = 3).

Applied individually, the warming stress had no significant effect on the levels of phototrophic community tolerance to Cu, as illustrated by the lack of significant difference (*p* > 0.05) between EC_50_ values obtained from toxicity tests on the photosynthetic efficiency of Control communities growing at 18, 23, and 28°C (**Figure 4A**). Whatever the tested temperature, the tolerance levels measured in Cu microcosms were higher than those measured in Control communities. However, significant differences (*p* < 0.05) were only detected at 23°C, due to a very high EC_50_ value obtained with Cu-exposed communities (283.8 ± 27.0 mg L^-1^)

##### Heterotrophic communities

The Cu tolerance assessment for heterotrophic communities was based on three extra-enzymatic activities (β-Glu, Lap, Pase). The short-term toxicity tests performed using Pase activity revealed no acute effect of Cu on this parameter in our experimental conditions (data not shown). Accordingly, no EC_50_ values were determined.

Applied individually, the warming stress had a significant influence (*p* < 0.05) on the tolerance levels estimated from toxicity tests on both β-Glu (**Figure 4B**) and Lap (**Figure 4C**) activities. However, the direction of temperature effects was reversed according to activity investigated. Results from the toxicity tests on β-Glu (**Figure 4B**) and Lap (**Figure 4C**) activities showed a gradual increase and a gradual decrease, respectively, in the tolerance of Control communities to acute Cu exposure when temperature increased, with significant differences between 18 and 28°C in both cases (*p* < 0.05).

Cu stress alone only had a significant effect on the tolerance estimated with β-Glu activity (**Figure 4B**, *p* < 0.05), leading to a 20-fold increase in EC_50_ values of Cu-exposed communities (1.02 ± 0.55 mg L^-1^) compared to Control communities (0.05 ± 0.03 mg L^-1^) under 18°C conditions. In contrast, Lap activity showed no significant difference at 18°C between Control (1.75 ± 0.19 mg L^-1^) and Cu-exposed (2.20 ± 0.12 mg L^-1^) communities, which were nevertheless slightly more tolerant to these metals (**Figure 4C**, *p* > 0.05).

The combined effect of warming and Cu on tolerance levels was also variable according to activity measured. There was no effect of temperature on EC_50_ values of Cu-exposed communities based on β-Glu activity measurements (**Figure 4B**, *p* > 0.05). Consequently, and due to the warming-induced increase in tolerance levels of Control communities based on β-Glu activity, there was no significant difference (*p* > 0.05) between Control and Cu-exposed communities at 28°C (in contrast to 18 and 23°C). Based on Lap activity, tolerance levels of Cu-exposed communities decreased with temperature, leading to a significant difference between 18 and 28°C (**Figure 4C**, *p* < 0.05) as observed with Control communities. However, this decrease was less pronounced in Cu-exposed communities (Lap EC_50_ = 1.44 ± 0.35 mg L^-1^ at 28°C) than in Control communities (0.64 ± 0.18 mg L^-1^ at 28°C). Consequently, there was a significant difference (*p* < 0.05) between tolerance levels of Control and Cu-exposed communities at 28°C (in contrast to 18 and 23°C).

## Discussion

### Influence of Experimental Warming on the Vulnerability of Phototrophic Communities to Cu Toxicity

In accordance with our first hypothesis, temperature had significant effects on the structure of diatom assemblages of Control communities. Taxonomic analysis revealed a shift in diatom assemblage composition with increasing temperature, as previously shown by Morin et al. (2017), thus leading to a strong decrease in diversity after 4-week growth at 28°C. This result was consistent with Larras et al. (2013) who also observed a decrease in diatom diversity in periphyton communities between 18 and 28°C. In our study, increasing temperatures selected *Fragilaria gracilis* (FGRA), which accounted for more than 95% of total diatoms at 28°C at the end of the experiment (week 4), suggesting that this species was particularly adapted to the temperature increase. Despite this marked shift in diatom assemblages, and contrary to Lambert et al. (2017), we found no significant effect of temperature on total algal biomass nor on photosynthetic efficiency. This was consistent with the findings of Pesce et al. (2009), who reported that effects of environmental stressors on microbial diversity (i.e., algal and bacterial composition) are not always detectable with community-level endpoints such as algal biomass and microbial activities, according to the concept of functional redundancy (Lawton and Brown, 1993).

Even more surprisingly, we did not find significant difference in the tolerance level of Control phototrophic microbial communities to Cu (based on EC_50_ estimated from photosynthetic yield measurement) after a 4-week growing period at 18, 23, and 28°C. These results diverge from Lambert et al. (2017) and Morin et al. (2017), who previously observed that experimental warming (from 18 to 28°C and from 8 to 23°C, respectively) significantly decreased the basal tolerance of phototrophic periphyton to acute Cu exposure. In these previous studies, changes in basal tolerance were potentially attributable to changes in phototrophic community structure as well as to a physiological stress induced by the temperature increase, which decreased photosynthetic activity and weakened the communities exposed to experimental warming. Here, the lack of significant effect of temperature on the photosynthetic efficiency of Control phototrophic communities suggests that the experimental warming did not drastically affect their physiological state. Moreover, the observed changes in structure of the Control phototrophic communities did not modify their vulnerability to acute Cu toxicity. This suggests that the temperature-induced species succession led to the replacement of the most temperature-sensitive species by species that are less temperature-sensitive but relatively similarly Cu-sensitive. Since the experimental warming mainly led to an increase in proportion of FGRA, which became strongly dominant at 23 and 28°C, to the expense of *Nitzschia palea* (NPAL) and *Achnanthidium minutissimum* (ADMI), it could be hypothesized that these three species exhibit comparable sensitivity to Cu. Both ADMI and FGRA (Morin et al., 2012a,b) were previously reported to be tolerant to metals. The tolerance of FGRA to Cu was confirmed here, since this species was well represented in Cu microcosms with mean proportions varying between 5.7 and 11.6% according to temperature tested. In addition, chronic Cu exposure mainly selected for NPAL, which was highly predominant in Cu-exposed communities whatever the temperature. Roussel et al. (2007) and Tien (2004) already highlighted that NPAL tends to be abundant in periphyton growing in metal-polluted waters. All these elements suggest that 4-week Control communities were mainly composed of relatively Cu-tolerant diatom species, whatever the temperature conditions. This could explain why EC_50_ values obtained in Control communities at the three tested temperatures (i.e., mean value higher than 3.0 mg L^-1^) were relatively high compared to EC_50_ values (i.e., 0.2–3.0 mg L^-1^) measured previously using the same toxicity test protocol with Control communities sourced from the same sampling site and grown for 3 weeks in the same experimental conditions (Lambert et al., 2017). Comparison of our results against Lambert et al. (2017) and Morin et al. (2017) finds that the influence of warming on the basal tolerance of phototrophic biofilm communities to Cu is strongly conditioned by the initial intrinsic characteristics of the community, especially initial distribution of temperature-sensitive (and temperature-tolerant) and/or Cu-sensitive (and Cu-tolerant) species and the temperature-induced succession.

Without temperature stress (i.e., at 18°C), the chronic Cu exposure delayed algal growth. Chl a concentrations were significantly lower in Cu-exposed than Control communities at week 2. Guasch et al. (2002) also observed growth inhibition with periphyton grown for 16 days at 16°C under comparable Cu exposure conditions (i.e., 10-17 μg L^-1^). At 18°C, Cu exposure also had significant effects on algal structure, with a shift in diatom assemblage composition, as shown in other studies (Serra et al., 2009; Morin et al., 2012b). At week 4, Cu exposure mainly selected for NPAL, which was about threefold more represented in Cu-exposed communities (i.e., 76%) than Control communities at 18°C. However, and despite the selection of this Cu-tolerant species at 18°C, no Cu-induced tolerance was found at phototrophic community level. Indeed, no difference in EC_50_ values based on photosynthetic yield was observed between Control and Cu-exposed communities at week 4, even if Cu-exposed communities appeared slightly more tolerant to acute Cu exposure. This lack of significant difference could be explained in the relatively high tolerance level observed in Control communities (see earlier) and by the moderate tolerance level of Cu-exposed communities (i.e., EC_50_ close to 5 mg L^-1^) which was far lower than EC_50_ values observed in previous studies (e.g., about 45 mg L^-1^ in Lambert et al., 2017). The lack of gain in Cu tolerance suggests a limited exposure of periphytic phototrophic microorganisms to Cu. This hypothesis is supported by the observation of a slight stimulation of the photosynthetic efficiency of Cu-exposed communities, at 18°C, over the study (with a significant difference compared to Control communities at week 4), which could reflect a hormesis effect (i.e., favorable biological response to low exposure to stressor), in contrast to previous studies where Cu exposure reduced biofilm photosynthesis efficiency (e.g., Serra and Guasch, 2009; Lambert et al., 2012). Cu analysis in periphyton also argues for this hypothesis, as periphyton communities were characterized by low Cu bioaccumulation, with total Cu concentrations reaching only 168–211 μg Cu g^-1^ dw, in contrast to Lambert et al. (2016) who found mean total Cu concentrations of 1580 μg Cu g^-1^ dw under similar temperatures (i.e., 18 and 23°C) and comparable dissolved Cu concentrations (i.e., 3–20 μg L^-1^). Moreover, Cu concentrations measured here in the intracellular and capsular fractions were almost fourfold lower than those in the corresponding fraction (i.e., so-called “internalized”) measured, at 18 and 23°C, in Lambert et al. (2016). The low Cu uptake by periphyton was in line with stable dissolved Cu concentrations measured in microcosms after each water renewal, contrary to previous microcosm studies showing a decrease in dissolved Cu concentrations due to high Cu uptake rates by periphyton (Knauer et al., 1997; Boivin et al., 2007; Lambert et al., 2012, 2016). Nevertheless, the lack of decreasing dissolved concentrations must be viewed with caution, since water sampling was performed 2 h after the addition of dissolved Cu. Indeed, Levy et al. (2007) reported that 1 h of Cu exposure was sufficient to ensure equilibrium between Cu in solution and Cu in marine algal cells. Accordingly, we cannot exclude that a decrease in Cu dissolved concentrations could have occurred immediately after Cu addition in the channels. The observed low Cu bioaccumulation could be explained by (i) high pH values (ranging between 8.3 and 9.7 during the experiment) which could induce a decrease in Cu bioavailability by favoring precipitated forms of Cu and/or by (ii) a strong biodilution of Cu in the periphyton biomass. This last hypothesis was supported by the fact that the periphyton biomass measured here at 18°C was a 1000-fold higher than measured at the same temperature by Lambert et al. (2016). Furthermore, this high periphyton biomass could have accelerated Cu adsorption, and prevented Cu internalization, due to an increase in the number of fixation sites in the EPS matrix.

Cu bioaccumulation, while being statistically non-significant, nevertheless tended to decrease with increasing temperature, as previously shown (Lambert et al., 2016) mainly because of a decrease in externalized Cu concentrations represented by colloidal and capsular fractions. It suggests that communities growing at 23 and 28°C under chronic Cu exposure were less exposed than those growing at 18°C. Nevertheless, and as observed with Control communities, temperature had no effect on photosynthetic efficiency of Cu-exposed communities. Furthermore, temperature combined with Cu had no effect on the structure of diatom assemblages, in contrast to Control communities, which suggests that Cu exposure was the main factor controlling the evolution of Control diatom assemblages. NPAL remained dominant whatever the temperature tested, showing that this species, which was favored by Cu exposure, was also particularly adapted to the temperature increase. In accordance with the observed stability in diatom assemblage structure, the tolerance levels of phototrophic Cu-exposed communities measured at week 4 were very similar at 18 and 28°C. However, and surprisingly, a strong increase in EC_50_ values was observed at 23°C. Based on our data, we cannot explain why this jump in tolerance occurred at this temperature. Note, however, that this jump occurred simultaneously with strong algal growth inhibition in Cu-exposed communities at 23°C, which could reflect a functional cost of adaptation (i.e., tolerance increase), as suggested by Navarro et al. (2008) who observed a reduction in chlorophyll a concentrations in biofilms which increased their tolerance to ultraviolet radiation.

### Influence of Experimental Warming on the Vulnerability of Heterotrophic Communities to Cu Toxicity

PICT analysis was performed on heterotrophic communities using three enzymatic activities, i.e., β-Glu, Lap, and Pase. The results obtained and the resulting interpretations varied greatly according to activity considered. As mentioned above, Cu had no acute toxicity on Pase activity, thus precluding any conclusions based on this heterotrophic parameter. Conversely, acute exposure to Cu strongly inhibited β-Glu and Lap activities. Analysis of the dose–response curves obtained with those enzymatic activities showed a strong influence of temperature on the sensitivity to Cu of Control heterotrophic communities. When considering β-Glu activity, we observed an increase in tolerance levels of Control communities with increasing temperatures, as proposed in our second hypothesis, which was based on the study of Boivin et al. (2005). Thus, based on this enzymatic activity, Control communities growing at 23 and 28°C appeared to be respectively about 8- and 18-fold more Cu-tolerant than those growing at 18°C. This suggests that the experimental warming increased the basal tolerance of heterotrophic periphyton to acute exposure to Cu, thus decreasing their vulnerability toward this toxic stress. This is in line with a field study by Faburé et al. (2015) who observed that Cu tolerance of natural biofilms (estimated from toxicity tests on the β-Glu activity) tended to increase during warm seasons.

Without temperature stress (i.e., at 18°C), and despite the limited Cu bioaccumulation in periphyton discussed above, heterotrophic communities chronically exposed to Cu exhibited significantly higher Cu tolerance levels than Control communities when bioassays were based on β-Glu activity measurements. This enzymatic activity has previously been successfully used to show an increase in Cu tolerance in PICT approaches in both experimental (Fechner et al., 2010; Tlili et al., 2010; Lambert et al., 2012) and *in situ* approaches (Fechner et al., 2012; Faburé et al., 2015). Contrary to the observation made with Control communities, the experimental warming had no influence on the tolerance levels of Cu-exposed communities obtained from the β-Glu activity. Accordingly, and given the fact that this tolerance level gradually increased with increasing temperature in Control communities, there was no difference in β-Glu EC_50_ values obtained at 28°C between Control and Cu-exposed communities. These results suggest that the chronic Cu exposure as well as the experimental warming led to a selection of heterotrophic microorganisms, which were more tolerant to Cu, revealing in this case a possible co-tolerance process.

Surprisingly, results obtained with the PICT approach using Lap activity came to the opposite conclusion. Indeed, in this case, the tolerance levels of Control communities strongly decreased with increasing temperatures, and were almost threefold lower at 28°C than at 18°C. The same trend was observed in Cu-exposed communities, even if the decrease in tolerance was more moderate, with an about 1.5-fold lower tolerance level at 28°C than at 18°C. Accordingly, it appeared from the PICT approach performed using Lap activity that the experimental warming increased the vulnerability of periphyton heterotrophic communities to acute Cu toxicity, whatever the previous exposure history to this metal.

In line with the PICT concept, chronic Cu exposure was also an important driver of the vulnerability of the Lap activity to subsequent acute exposure to Cu. Indeed, whatever the temperature tested and despite a lack of statistical difference at 18°C, the tolerance levels measured from Lap analysis where higher with Cu-exposed communities than with Control ones. Contrary to β-Glu activity, increasing temperature increased the difference in sensitivity to acute Cu toxicity between Control and Cu-exposed communities, revealing a lack of any co-tolerance process based on the Lap activity.

In summary, we demonstrated using three functional parameters (photosynthesis, Lap, and β-Glu) that the influence of temperature on the vulnerability of phototrophic and heterotrophic microbial communities to Cu toxicity can vary greatly according to function considered. A similar conclusion was previously reached by Tlili et al. (2010) who found that the relative influence of chronic exposure to Cu and phosphorus, respectively, on functional vulnerability of microbial communities to subsequent Cu exposure depended on the function studied (i.e., photosynthesis, substrate-induced respiration, Lap, and β-Glu). Taken together, these results argue that it is crucial to consider different functional compartments and different functional descriptors in order to better assess the vulnerability of periphyton to multiple stressors. From an ecotoxicological standpoint, it should be interesting to explore whether our findings are specific of Cu or whether they are applicable to other metals. Tlili et al. (2011) observed that phototrophic and heterotrophic biofilm communities exposed to Cu were more tolerant to zinc (Zn) and *vice versa* (with Cu being more toxic than Zn). It reflects a positive co-tolerance between these metals, which was attributed by the authors to similar modes of action and/or detoxification. Accordingly, we should expect that the influence of temperature on the vulnerability of communities to Cu and Zn (or others metals with similar modes of action and/or detoxification) toxicity would have been quite similar. In contrast, this hypothesis is probably not applicable with metals exhibiting a different mode of action or involving different detoxification pathways, such as arsenic (As). From an ecological standpoint, and given the fact that the functions studied are related to different biogeochemical cycles (i.e., photosynthesis and β-Glu: carbon cycle; Lap: nitrogen cycle), this kind of multi-function approach could allow to better assess and predict the risks of multiple stressors for ecosystem balance and functioning. Moreover it should be important to validate *in situ* the hypotheses derived from such experimental studies by performing field-based studies at large geographical and/or temporal scale.

## Author Contributions

SP, A-SL, MC, and AD conceived and designed the study. A-SL performed the experiments and samplings. A-SL, AF, SM, and AD analyzed the samples and all co-authors analyzed and interpreted the data. SP and A-SL drafted the article. AF, SM, MC, and AD critically revised the article. All co-authors approved the final submitted version of the manuscript.

## Conflict of Interest Statement

The authors declare that the research was conducted in the absence of any commercial or financial relationships that could be construed as a potential conflict of interest.
